# Dual-tasking modulates movement speed but not value-based choices during walking

**DOI:** 10.1038/s41598-024-56937-y

**Published:** 2024-03-15

**Authors:** Eric Grießbach, Philipp Raßbach, Oliver Herbort, Rouwen Cañal-Bruland

**Affiliations:** 1 Department for Neurology, Johns Hopkins University, Baltimore, MD USA; 2 Department for the Psychology of Human Movement and Sport, Friedrich Schiller University, Jena, Germany; 3 Department of Psychology, Julius-Maximilians-Universität Würzburg, Würzburg, Germany

**Keywords:** Multitasking, Decision-making, Motor control, Motor cost, Embodied decisions, Value-based decision-making, Human behaviour, Decision

## Abstract

Value-based decision-making often occurs in multitasking scenarios relying on both cognitive and motor processes. Yet, laboratory experiments often isolate these processes, thereby neglecting potential interactions. This isolated approach reveals a dichotomy: the cognitive process by which reward influences decision-making is capacity-limited, whereas the influence of motor cost is free of such constraints. If true, dual-tasking should predominantly impair reward processing but not affect the impact of motor costs. To test this hypothesis, we designed a decision-making task in which participants made choices to walk toward targets for rewards while navigating past an obstacle. The motor cost to reach these rewards varied in real-time. Participants either solely performed the decision-making task, or additionally performed a secondary pitch-recall task. Results revealed that while both reward and motor costs influenced decision-making, the secondary task did not affect these factors. Instead, dual-tasking slowed down participants’ walking, thereby reducing the overall reward rate. Hence, contrary to the prediction that the added cognitive demand would affect the weighing of reward or motor cost differentially, these processes seem to be maintained at the expense of slowing down the motor system. This slowdown may be indicative of interference at the locomotor level, thereby underpinning motor-cognitive interactions during decision-making.

## Introduction

Many daily decision-making situations entail a complex interplay between cognitive and motor processes^1^. That is, value-based choices can entail both action-independent (i.e., abstract) and action-dependent considerations, such as weighing the health benefit and motor cost of running instead of taking the car, respectively. Additionally, these considerations are often embedded within complex multitasking environments where multiple, cognitively demanding processes run in parallel with motor processes (e.g., talking to a friend or looking at the traffic while running or driving a car, respectively).

However, while both types of considerations are abundant for decision-making in real-life multitasking environments, current approaches often treat these processes in isolation. Cognitive decision-making research focuses predominantly on abstract choices while motor control research focuses on action-dependent decisions. This separation leaves a significant gap in our understanding of how the two core processes interact in more complex scenarios^2^.

Embodied decision theory^3–6^ attempts to bridge this gap by asserting that decision-making is not solely a cognitive process but is also fundamentally influenced by the body's physical and motor capabilities. It suggests that the body's state and potential actions directly impact cognitive processes involved in decision-making. While this perspective sheds light on the impact of physical actions and motor costs on cognitive evaluations in value-based decisions, it has yet to fully address how these processes interact in multitasking environments that involve an additional cognitive load.

The distinction between the roles of action-independent rewards and action-dependent motor costs in decision-making becomes particularly crucial in this context. Empirical evidence indicates that these two types of considerations may play distinct roles in value-based decision-making scenarios. For instance, choosing between rewards seems prone to cognitive disruptions. Amongst others, this is evidenced by studies showing that simultaneous engagement in a secondary task can disrupt decision-making^7,8^. The applied secondary tasks are typically designed to tax capacity-limited cognitive processes (e.g., working memory), which are simultaneously needed and hence reduced for decision-making processes^9^. These and similar findings indicate that weighing rewards is a capacity-limited cognitive process^10,11^. By contrast, the execution of skilled motor processes is often assumed to be automatic and independent of cognitive capacities^12^. In line with this conjecture, empirical work indeed shows that multitasking rarely interferes with skilled motor behavior, and if it does, it may even improve motor performance. For example, a study by Beilock, et al.^13^ found that experienced golfers and soccer players performed better under dual-task conditions in well-practiced motor tasks. This supports the idea that well-learned skills do not require or are even hampered by constant cognitively demanding control^13–17^.

This differential effect of multitasking on weighing rewards, on the one hand, and motor cost processing, on the other hand, extends to the temporal domain. While motor costs influence decision-making even at short timescales^18^, reward processing tends to be slower. This is highlighted by a recent study investigating the temporal dynamics of both reward and motor cost on decision-making. The influence of motor costs tends to predominate in the early timescales of the decision process^19^. With more decision time, though, the effect of rewards progressively dominates the decision process. Similar temporal dynamics can be observed in habitual versus goal-directed actions. Habits predominate at early timescales, but with more time they become progressively dominated by goal-directed movements^20^. Notably, habitual behavior is often found to be unaffected by multitasking, that is, to be automatic^21^. The mirroring of the time course of weighing motor costs versus reward and habitual versus goal-directed behavior seems to lend further support to the notion that the weighing of motor costs may be rather habitual and automatic^19^.

**Figure 1:**
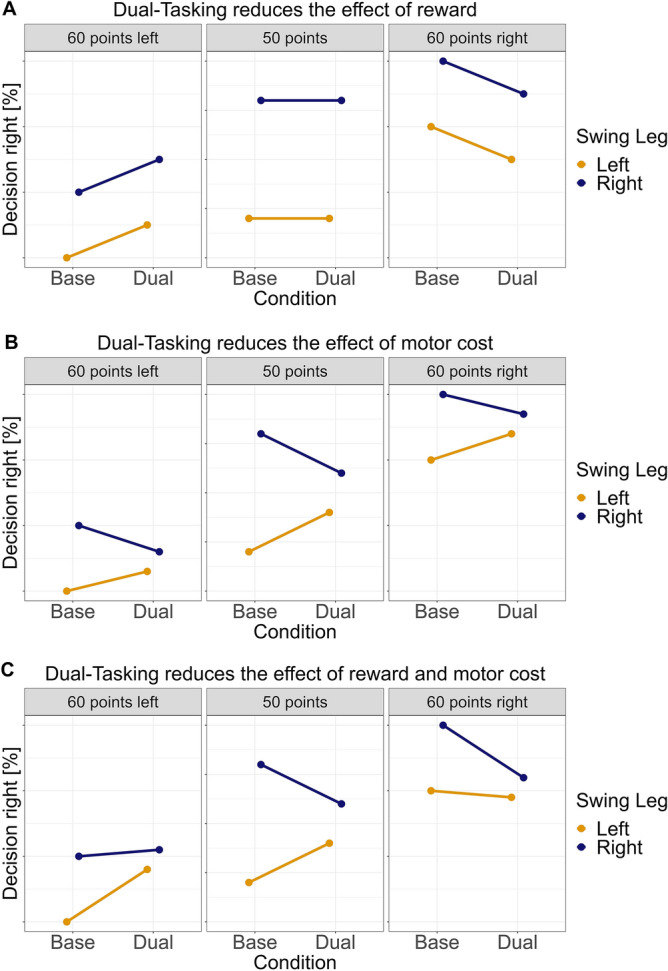
Qualitative illustration of hypothesized outcomes. The figure presents the expected results based on how the cognitive dual-task impacts decision-making processes. (**A**) Outcomes predicted if dual-tasking predominantly impairs the influence of reward, aligning with our primary hypothesis. (**B**) Outcomes predicted if dual-tasking primarily impairs the influence of motor cost, with notable differences in the equal reward (50/50 point) condition. (**C**) Outcomes predicted if dual-tasking impairs both the influence of reward and motor cost, i.e., making decisions generally more random.

Taken together, evidence suggests that the cognitive weighing process of rewards is slow and limited, for instance, by working memory capacities, whereas the embodied weighing of motor cost is faster and rather independent of working memory load. Assuming these processes are separate, we hypothesize that a secondary task taxing working memory predominantly impairs reward processing but should not affect (or—if anything—increase) the impact of motor costs (see Fig. 1A). Alternatively, embodied decision-making models argue that the influences of motor and cognitive processes are heavily intertwined and inseparable. Under this framework, a dual-tasking scenario is predicted to adversely affect both motor and cognitive influences on decision-making.

**Figure 2:**
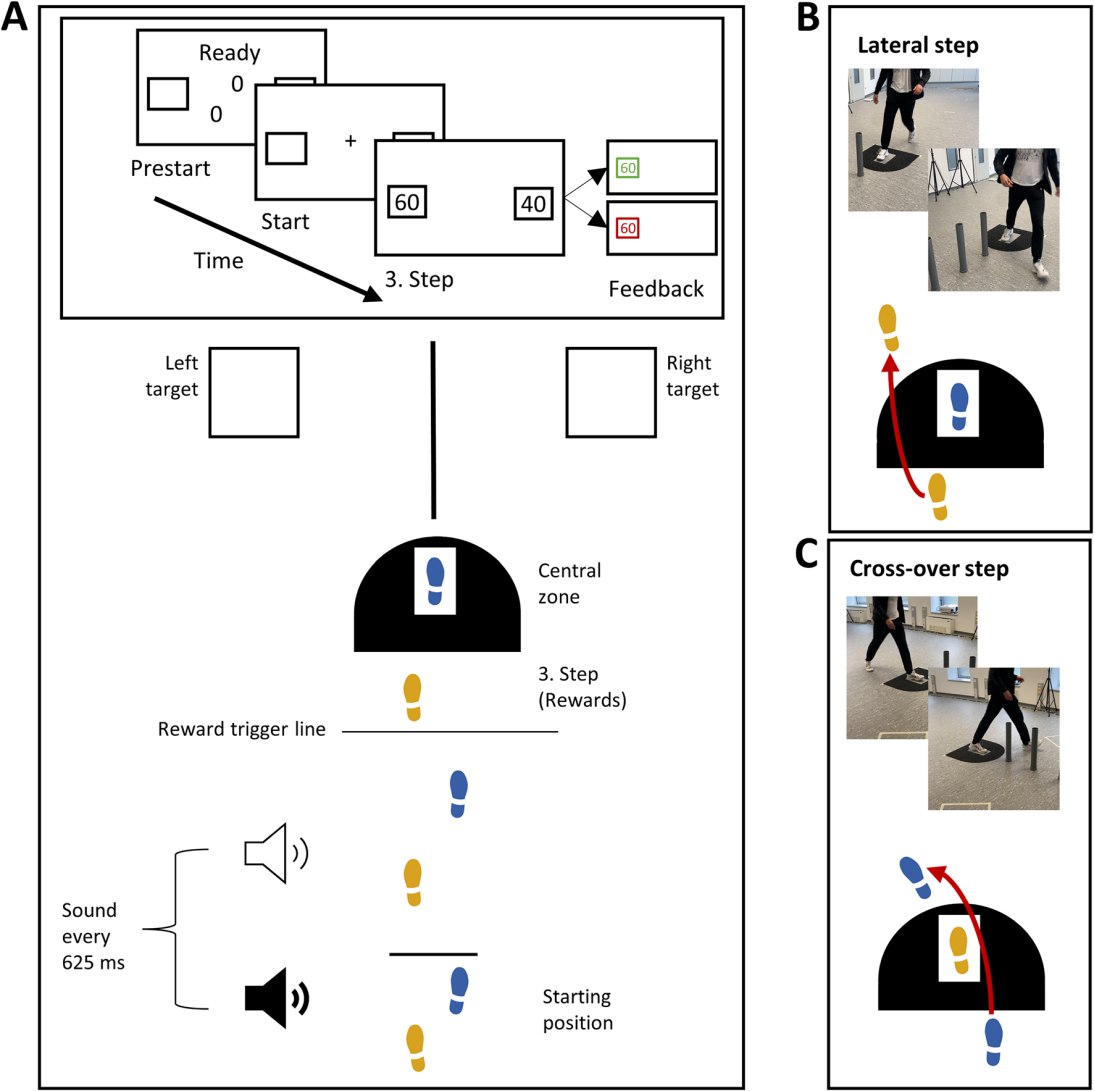
Experimental setup. (**A**) The upper part of the figure displays the information on a screen for the participant at various times during the trial. After assuming the starting position (here right foot in front), a “+” on the screen indicated the beginning of a trial and participants started to walk. Upon walking toward the central zone, a hidden, calibrated line—referred to as the "Reward trigger line"—triggers the display of rewards upon the next touch-down. Typically activated on the third step, this mechanism is set to reveal the rewards one step prior to entering the central zone. To receive either reward, participants had to walk into the central zone and bypass the obstacle to the left or right target, respectively. A high- or low-pitched sound was playing every 625 ms during the trial. For the baseline condition, the sounds should be ignored. In the dual-task condition, participants were instructed to memorize the sequential positions of high-pitch sounds and were asked to report them verbally after the trial. (**B**) Example of a lateral step, where the left swing leg is placed towards the left side. (**C**) Example of a cross-over step, where the right swing leg must cross the stance leg to be placed towards the left side.

In designing our study, we aimed to explore the weighing process of reward and motor cost in decision-making under varying cognitive loads. To achieve this, we employed a walking task, which we chose for its ecological validity, reflecting real-world decision-making scenarios such as navigating while walking or making split-second decisions in sports. This task also aligns with our previous research, where it has reliably produced both an influence of reward and motor cost on decision-making^22,23^. Specifically, participants were instructed to collect rewards by walking towards a left or right lateral target (see Fig. 2). Rewards were displayed while walking, just before reaching a central obstacle. During walking, the motor cost to walk to either target varied based on the side of the current swing leg. The side of the swing leg was manipulated by predetermining the starting position in each trial. Previous studies have shown that not only reward influenced participants’ decisions, but also their swing leg in accordance with the varying motor costs^22^, a phenomenon which we will call swing leg effect (SLE) in the remainder of the paper.

Additionally, to study whether the weighing of reward and motor cost is limited by cognitive resources, we introduced a dual-task condition alongside a baseline condition. The secondary task required participants to memorize the sequential order of high versus low-pitched tones played during the trial. Thus, the auditory task was expected to increase the cognitive load without interfering with the visual processing of rewards^24^. A similar secondary task was used in prior studies to create cognitive crosstalk^14,25^. We hypothesized that if the weighing of reward is dependent on capacity-limited cognitive processes, but the weighing of motor cost is automatized and both processes are separable, the decision should be less dependent on the reward and equally or more dependent on the current swing leg under the cognitive load of the secondary task (see Fig. 1A). If, however, the processes are inseparable and the decision process per se is capacity-limited, we would expect to see qualitatively different outcomes, with both reward and the swing leg effect adversely affected by dual-tasking (as illustrated in Fig. 1B,C).

## Methods

### Participants

The study involved a total of 39 participants, with the sample size relying on prior experiments with a similar design in which we reliably found an influence of reward and motor cost on decision-making^22,23^. Two participants were excluded for the final analysis. One individual consistently utilized the same foot when entering the central zone, making their contribution to the estimation of the swing leg effect (SLE) negligible. Another participant was not reliably able to solve the secondary task (see supplementary results). Hence, the final sample size consisted of 37 participants (15 female, 22 male, age: mean = 23.0 years, sd = 2.6 years, handedness: 36 right-handed, 1 left-handed, footedness: 34 right-footed, 1 left-footed, 2 no preference). Upon completion of the experiment, each participant received a predetermined payment of 15.00 €. All participants provided written informed consent. The experiment conducted in this study was approved by the ethics committee of the Faculty of Social and Behavioral Sciences at Friedrich Schiller University Jena. All research was performed in accordance with the relevant regulations and in accordance with the Declaration of Helsinki. All participants gave an informed consent before the start of the experiment.

### Apparatus and stimuli

Figure 2A shows the setup of the task. The setup included a central zone and two targets, one to the left and one to the right. Each target was placed at a 52.5° angle and 1.5 m away from the central zone. These targets were 0.5 m squares. The central zone itself was a smaller rectangle, measuring 0.35 m by 0.2 m. Surrounding the central zone, there was a semicircle carpeted area with a radius of 0.5 m, and a small rectangular extension of 0.15 × 0.5 m to constrain the step behavior. Additionally, three cylindrical pipes (3.7 cm in radius and 55 cm in height) served as obstacles. These were positioned 0.6 m behind the center of the central zone, evenly spaced with a 0.3 m gap between each, effectively dividing the left and right sides. The setup, including the starting line, central zone, and lateral targets, was demarcated using black tape.

To capture participants' gait behavior, we used a 3D infrared motion capture system consisting of eight Prime 17W cameras (Optitrack, Corvallis, US). This system, operating at a frequency of 120 Hz, tracked movement through passive reflective markers, each 12 mm in diameter. These markers were placed on specific locations on the participants' feet: the lateral malleolus, heel, and between the second and third metatarsal heads on both feet.

A 27-inch screen on a desk centrally positioned behind the obstacles (2.560 × 1.440 resolution; 60 Hz frame rate; Lenovo P27h; Lenovo; Lenovo Beijing China) was used to display all stimuli. Stimuli consisted of black, green, or red elements on a white background and were dynamically generated in real-time using a custom MATLAB script (refer to the Data Analysis section for more details). To do that, marker positions were streamed using the NatNet SDK, interfacing with Motive v2.1.1 (Optitrack software), to the MATLAB script (MATLAB 2018a The Mathworks, Inc., Natick, MA, USA). Key events included the initiation of a trial, the kinematically determined step^26^ after reaching the reward trigger line, the step in the central zone, and the end of a trial (see Supplementary Information, SI, for further details on event detection). The distance of the start position and the individual time limit for each participant were predetermined based on their baseline walking patterns observed prior to the experiment (see SI). Auditory feedback was given by an external 2.1 sound speaker system (Wavemaster Cube Mini, Bremen, Germany) connected to the PC at a sampling rate of 48,000 Hz.

### Procedure

Prior to experimentation, participants were informed about the experimental procedure, completed several questionnaires (demographic data; Edinburgh Handedness Inventory for handedness^27^; lateral preference inventory for footedness^28^) and gave written informed consent. Subsequently, reflective markers were placed on the participants, and calibration trials were conducted to ascertain the individual starting distance and trial duration. During the calibration trials, participants were instructed to walk to the other side of the room as quickly as possible, with the initial four steps' time and distance determining the starting position and the time limit required to complete a trial for a higher reward. Like in a prior study^22^, we determined the individual time constraint as the time between the initiation of the gait movement and the touchdown of the fourth step, plus a fixed time constant of 2.1 s to account for reaction time and the time required to get from the central zone towards the targets (see SI for a more detailed description).

Following the calibration trials, participants were presented with experimental instructions through a pre-recorded, narrated PowerPoint presentation. The instructions outlined the following key points: Participants were instructed to walk from a designated starting line either to a left or right target for a reward. The starting position of their feet was displayed on a screen (e.g., left foot forward or right foot forward). Prior to approaching the target, participants had to enter the central zone situated in front of an obstacle. Rewards for each target were visible on a screen located behind the obstacle as the participants advanced. Three different reward combinations were possible: 60/40, 50/50, and 40/60 points for the left and right side, respectively.

To receive the higher reward, participants needed to complete the trial within the allotted time for the corresponding target. Time-related feedback was provided on the screen during certain training trials, and participants were instructed to maximize their reward rate. Performance feedback was displayed in red (too slow) or green (within time) after each trial.

During a trial, a low (500 Hz) or high (1000 Hz) sound played for 250 ms every 625 ms after the trial's initiation until its conclusion. The sound's pitch was randomly selected, with a 1/3 chance of being high and a 2/3 chance of being low. In the dual-task condition, participants were required to attend to the pitch throughout the trial and subsequently recall the temporal position of the high-pitched sounds in the sequence (e.g., for a high/low/low/low/high sequence, they had to report positions 1 and 5). Conversely, in the baseline condition, participants were instructed to disregard the sounds.

Participants completed two blocks of trials, one in the dual-task condition and the other in the baseline condition, with the order counterbalanced across participants. Each participant underwent 18 familiarization trials and 120 experimental trials, divided into two blocks. Before the first block, the experiment commenced with six familiarization trials without dual-tasking, and feedback was provided regarding trial duration in seconds. This was followed by six familiarization trials in the respective starting condition of the experiment (baseline or dual tasking), with binary feedback about trial duration (finished in time or not) and 60 experimental trials. Prior to starting the remaining 60 experimental trials, another six additional familiarization trials were conducted in the respective condition.

The experimental phase consisted of a 2 (dual-tasking: yes or no) × 2 (starting position: left/right foot at starting line) × 3 (point combination left/right: 40/60, 50/50, 60/40) × 10 trial design, with trials presented in random order. The entire task took approximately 60 min to complete.

### Data analysis

In this study, stimuli were dynamically presented in response to the participant's movements, tracked via markers. We used the NatNet SDK in conjunction with Motive v2.1.1 (OptiTrack software interface) to stream the 3-D positions of these markers to a custom MATLAB 2018a script (The MathWorks, Inc., Natick, MA). The initiation of a trial, the timing for reward cues just before the central zone was reached^26^, the moment of stepping into this zone, and the trial's conclusion were all determined by the location of the foot markers (detailed methods are provided in the SI).

For subsequent analysis, we applied a bidirectional fourth-order low-pass Butterworth filter with a 12 Hz cutoff to the kinematic data. Missing values were interpolated for up to 25 frames (equivalent to 0.21 s) using cubic spline interpolation. To verify the real-time data from the experiments, trials that raised concerns were individually examined based on their kinematic profiles (our methods for identifying these trials are outlined in the SI). After visual inspection of these trials, 4301/4560 trials (94%) were included in the statistical analysis. One participant stopped the experiment early and missed 16 and 24 out of 60 trials for the baseline and the dual-tasking condition, respectively. 219 trials were excluded because rewards were displayed too late or because of technical problems (e.g., dropping a marker during the trial or tracking errors).

For the statistical analysis, we used R^29^. To investigate the influence of swing leg (left or right), reward combination (60/40, 50/50, and 40/60 for the left/right side), and dual-tasking (baseline/dual-task) on participants’ decisions (left/right side) we used a generalized linear mixed model^30^ with a Bayesian approach^31,32^. We opted for a mixed model to account for the multiple measures of binary decisions within participants which leads to non-independent data^30^. We chose to utilize a Bayesian statistical approach, as it is considered more robust for fitting Generalized Mixed Models^32^. Model fitting was done with the brms package^33^. For the analysis and reporting we followed the guidelines of Kruschke^34^. Our scripts and data are publicly shared in a OSF repository, see https://osf.io/V85D7/.

In analyzing decision outcomes (choices between left or right), we employed a Bernoulli distribution, coupled with a logit link function. Our specifications for contrasts were grounded in our a priori hypothesis^35^. To examine the influence of varying rewards, we utilized a Helmert contrast. This contrast allowed us to assess the effect of different reward levels (e.g., comparing 40 points on the right vs. 60 points on the right) and to contrast unequal reward scenarios with equal reward conditions (the average of 40 and 60 points on the right vs. 50 points on the right). For analyzing the influence of the swing leg and dual-task conditions, we implemented a centered sum contrast. This contrast was designed to compare the effects of the right swing leg and baseline condition (coded as 0.5) against the left swing leg and dual-tasking condition (coded as − 0.5). To account for inter-subject variability, our model included a random intercept and all relevant random slopes^36^, but we excluded correlation parameters between random effects as they do not influence estimations of fixed effects but increase model complexity and the resulting computation time^32,36^. Full details on the prior distributions used are available in the Supplementary Information section. The specific formula used in our R script for this model is detailed below:

$${\text{Right}}\left| {{\text{trials}}\left( {{\text{Trial}}} \right) \sim {\text{Points}}\_{\text{R}}*{\text{Swing}}\_{\text{Leg}}*{\text{Dual}}\_{\text{Tasking}} + ({\text{Points}}\_{\text{R}}*{\text{Swing}}\_{\text{Leg}}*{\text{ Dual}}\_{\text{Tasking }}} \right||{\text{Subject}})$$

In the Bayesian analysis, each parameter is represented by a posterior distribution. This distribution reflects the range of potential parameter values, integrating prior knowledge, the observed data likelihood, and the overall model structure. To succinctly describe these posterior distributions, we present several key summary statistics. These include the estimated mean of the distribution, the 95% credible intervals (CrIs) calculated using equal-tailed intervals, and the probabilities of the parameter values exceeding or falling below certain predefined thresholds. Parameters that exhibit a greater than 95% probability of differing from opposite signed effects are explicitly highlighted in the text. We also provide Bayes Factors for evaluating the Null-Model and Evidence Ratios for evaluating directional hypothesis. The Bayes Factor (BF) quantifies the strength of evidence for one model over another, updating our prior beliefs into posterior probabilities. Specifically, a BF_01_ value of 10 implies that the null hypothesis (β = 0) has become ten times more likely given the observed data, relative to the alternative model (β ≠ 0). In addition to Bayes Factors, we employ Evidence Ratios to evaluate directional hypotheses. These ratios represent the likelihood of the data under models with contrasting signs for β, effectively comparing the posterior probabilities for β > versus β < 0.

### Usage of large language models

In refining this manuscript, the authors employed ChatGPT^37^ to enhance the conciseness of the text. It is important to clarify that ChatGPT's role was strictly limited to rephrasing existing sentences and sections; it did not contribute any novel content. The original ideas and information remain solely the work of the authors.

## Results

**Figure 3:**
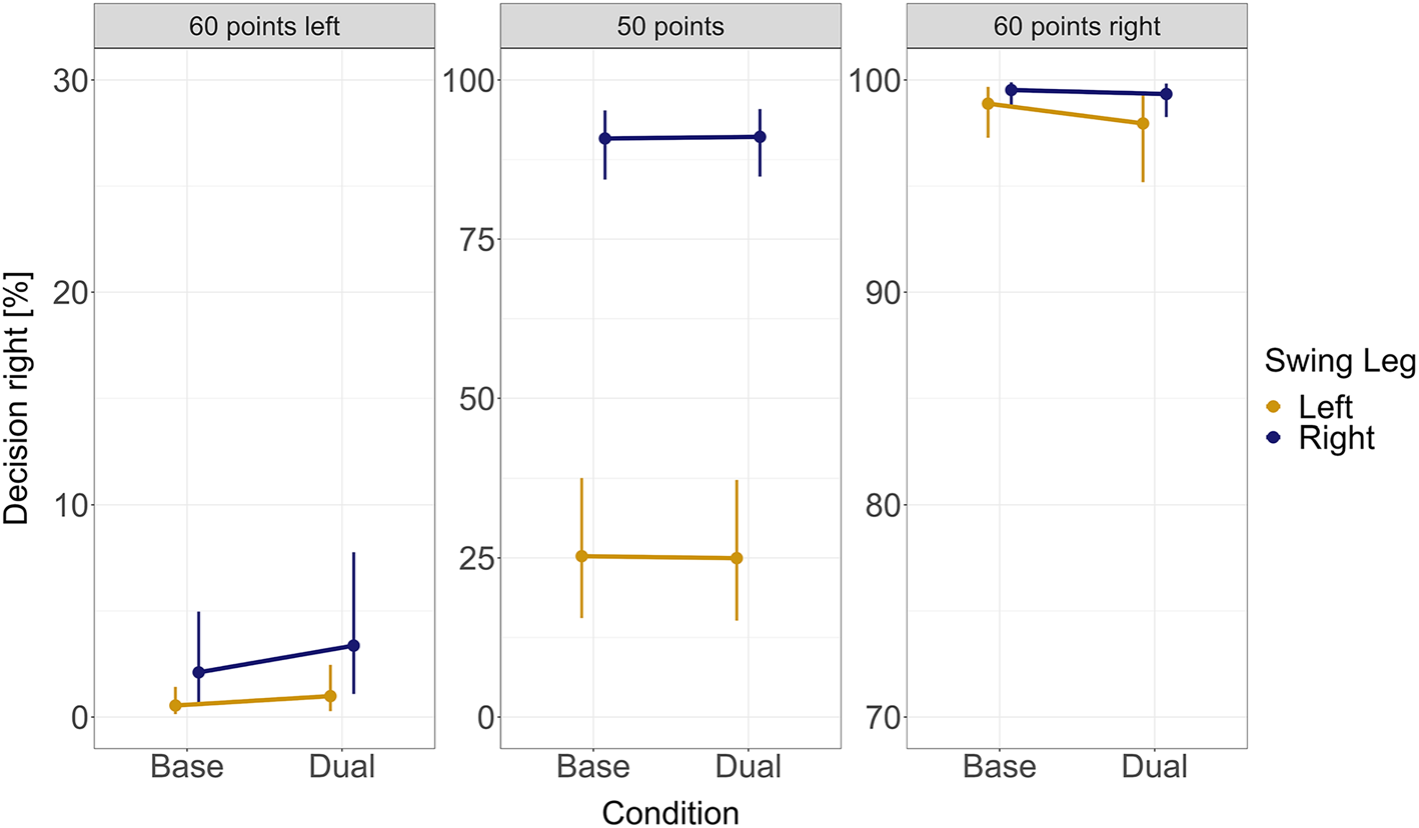
Empirical results for the effect of reward, the swing leg, and dual-tasking on decision-making. Displayed are summary statistics of the posterior distribution (mean and 95% CrI) after fitting the model to the data. Note that for clarity reasons the y-scale is different for the three plots.

The statistical analyses of the effects of reward, swing leg, and dual-tasking on decision-making are summarized in Table 1. This table presents values derived from a test scale that linearizes probability data into log-odds ratios. Additionally, Fig. 3 displays the conditional estimates (mean and 95% CrI) of the empirical data. In the text, we will highlight statistical comparisons testing main hypotheses.

**Table 1:** Statistical estimates for decision-making analysis.

Effect	β	95% CrI	p(β = 0)	BF_01_	p(β > 0)	ER_+_
Intercept	0.36	− 0.02 to 0.75	0.48	0.92	0.97	32.08
RE	**4.62**	**3.87 to 5.41**	** < 0.001**	** < 0.001**	** > 0.999**	** > 100**
RE-Equal	0.13	− 0.01 to 0.27	0.54	1.16	0.97	32.29
SLE	**1.95**	**1.27 to 2.57**	** < 0.001**	** < 0.001**	** > 0.999**	** > 100**
Dual-Task	0.03	− 0.36 to 0.40	0.72	2.52	0.56	1.25
RE:Dual-Task	− 0.52	− 1.13 to 0.11	0.28	0.39	0.05	0.05
DT:RE-Equal	− 0.01	− 0.23 to 0.21	0.82	4.69	0.47	0.89
RE:SLE	− 0.15	− 0.61 to 0.31	0.63	1.72	0.26	0.35
RE-Equal:SLE	**0.76**	**0.47 to 1.06**	** < 0.001**	** < 0.001**	** > 0.999**	** > 100**
DT:SLE	0.07	− 0.51 to 0.63	0.63	1.68	0.59	1.45
DT:RE:SLE	0.21	− 0.47 to 0.88	0.55	1.20	0.73	2.66
DT:RE-Equal:SLE	− 0.01	− 0.37 to 0.36	0.73	2.74	0.48	0.94

This table presents a summary of each model parameter, including the mean estimate on the log-odds scale (β), the 95% credible interval (CrI), the probability that the estimate is zero, the Bayes Factor (BF_01_), the probability that the posterior is greater than zero, and the evidence ratio of the parameter being positive rather than negative (ER_+_). Parameters with a high probability of being smaller or greater than 1 are highlighted with a bold font (< 0.05 or > 0.95). For contrasts see methods. Model formula: logit(p_Side_) ~ Points_R * Swing_Leg * Multitasking + (Points_R * Swing_Leg * Multitasking||Subject).

*RE* = reward effect, *SLE* = swing leg effect.

### Reward and the swing leg affected decision-making

Similar to previous findings^22,23,38^ decision-making was affected by reward. Participants dominantly went to the side displaying 60 points (see Fig. 3). The likelihood of choosing the right side was conclusively higher if 60 points were on the right side vs. on the left side (β = 4.62, 95% CrI = 3.87 to 5.41, p(β = 0) < 0.001, BF_01_ = 0.001, p(β > 0) > 0.999, ER +  > 100).

Additionally, participants were influenced by the swing leg before turning towards either target. Participants walked more often to the right side, given a right swing leg compared to a left swing leg (β = 1.95, 95% CrI = 1.27 to 2.57, p(β = 0) < 0.001, BF_01_ < 0.001, p(β > 0) > 0.999, ER_+_  > 100). The SLE was moderated by the reward condition (β = 0.76, 95% CrI = 0.47 to 1.06, p(β = 0) < 0.001, BF_01_ < 0.001, p(β > 0) > 0.999, ER_+_  > 100), indicating that the SLE was stronger for the equal reward condition (50 points left and right; β = 3.47, 95% CrI = 2.66 to 4.23, p(β = 0) < 0.001, BF_01_ < 0.001, p(β > 0) > 0.999, ER_+_  > 100) compared to the unequal reward condition (60 points left or right vs. 40 points; β = 1.20, 95% CrI = 0.40 to 1.95, p(β = 0) = 0.02, BF_01_ = 0.02, p(β > 0) = 0.998, ER_+_  > 100). That means that there is overwhelming evidence for a swing leg effect for equal rewards, and also strong evidence for a (weaker) swing leg effect for unequal rewards.

### Dual-tasking did not reliably affect decision-making

Participants showed significantly greater accuracy in identifying sound pitches in the secondary task than would be expected by chance (p < 0.01; see SI). This indicates active engagement with the secondary task. Despite the attendance, dual-tasking did not have a reliable impact on decision-making (see Fig. 3). There was only a tendency that the reward effect decreased under dual-tasking (β = − 0.52, 95% CrI = − 1.13 to 0.11, p(β = 0) = 0.28, BF_01_ = 0.39, p(β > 0) = 0.05, ER_+_  = 0.05). Similarly, dual-tasking did not reliably alter the swing leg effect (β = 0.07, 95% CrI = − 0.51 to 0.63, p(β = 0) = 0.63, BF_01_ = 1.68, p(β > 0) = 0.59, ER_+_  = 1.45).

### Dual-tasking affected the time to finish a trial and the overall reward rate

**Figure 4:**
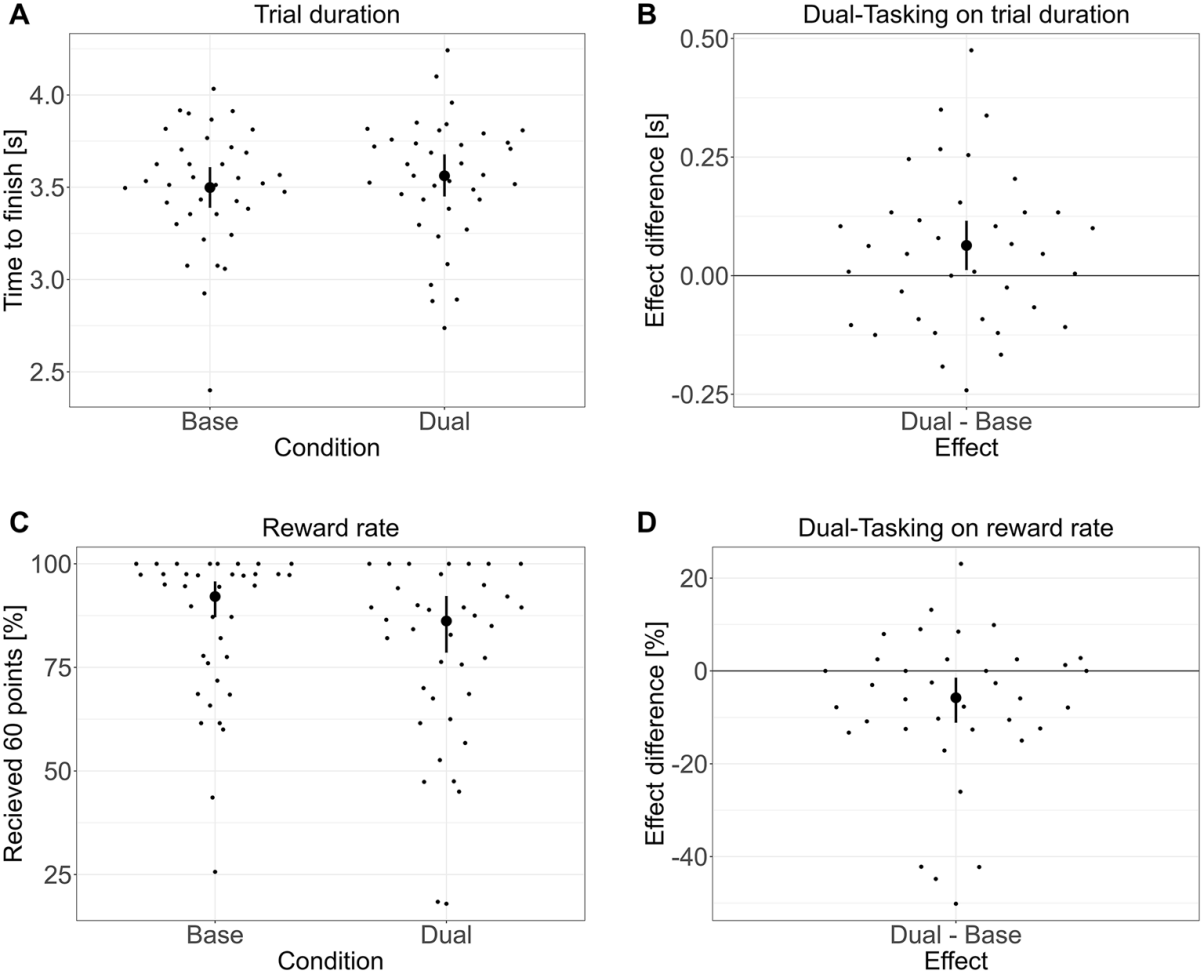
Impact of dual-tasking on overall trial completion time and reward acquisition rate. Displayed are summary statistics of the posterior distribution (mean and 95% CrI) after fitting the model to the data. The left-side plots present conditional estimates, while the right-side plots display the difference between baseline and dual-tasking conditions (difference plots). Unlike the Reward Effect (RE), which focuses solely on the chosen destination, the Reward Rate here considers both the choice and timely trial completion. (**A**) Conditional estimates (mean and 95% CrI) for completion time under single and dual-task conditions. (**B**) Difference plots illustrating the change in completion time due to dual-tasking. (**C**) Conditional estimates for reward probability with and without multitasking. (**D**) Difference plots showing the change in reward probability due to dual-tasking. Bold dots display average model estimates and their 95% CrI, while scattered dots represent individual participants’ median values.

While dual-tasking did not reliably affect target choices, it increased the time to finish a trial (β = 0.064 s, 95% CrI = 0.012 to 0.116 s, p(β = 0) = 0.52, BF_01_ = 1.10, p(β > 0) = 0.991, ER_+_  = 114.52, see Fig. 4). The increased time to finish also impacted the reward rate, resulting in participants finishing more frequently outside the time limit. That is, the probability of receiving 60 points was reduced with dual-tasking (β = − 0.63, 95% CrI = − 1.08 to − 0.17, p(β = 0) = 0.06, BF_01_ = 0.07, p(β > 0) = 0.004, ER_+_  = 0.004, see Fig. 4).

**Figure 5:**
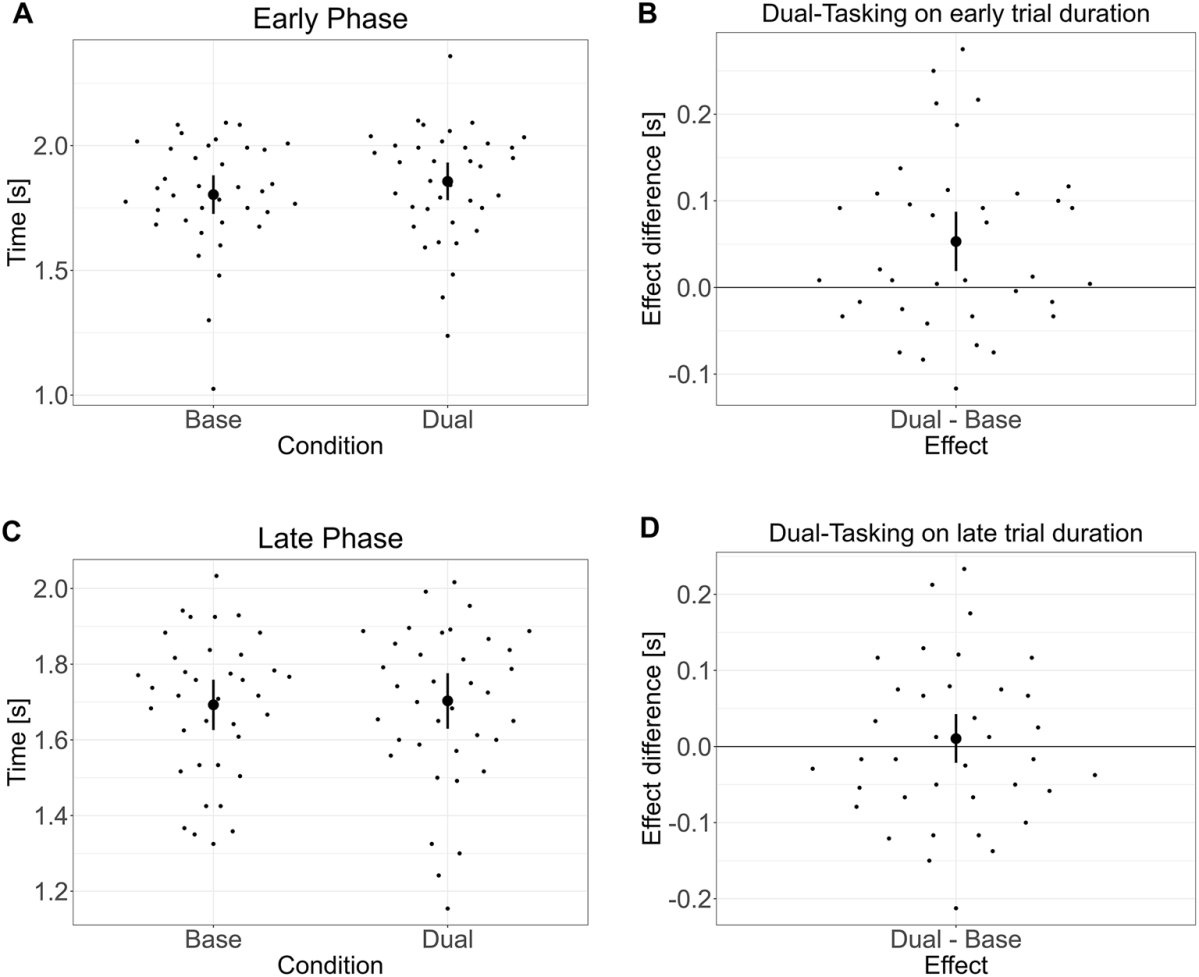
Impact of dual-tasking on overall trial completion time dependent on the phase within a trial. Displayed are summary statistics of the posterior distribution (mean and 95% CrI) after fitting the model to the data. The left-side plots present conditional estimates, while the right-side plots display the difference between Baseline and Dual-Tasking conditions (difference plots). The early phase spans from the trial’s start to the display of rewards, while the late phase covers the time from reward display to trial end. (**A**) Conditional estimates of phase duration under single and dual-task conditions. (**B**) Difference plots showing the change in phase duration due to dual-tasking. (**C**) Conditional estimates for reward probability with and without multitasking. (**D**) Difference plots illustrating the change in reward probability due to dual-tasking. Bold dots highlight average model estimates and their 95% CrI, and scattered dots represent the median values for individual participants.

A more detailed explorative analysis indicated an interaction between an increased time early vs. late in the trial (β = 0.043 s, 95% CrI = 0.005 to 0.080 s, p(β = 0) = 0.69, BF_01_ = 2.27, p(β > 0) = 0.986, ER_+_  = 67.77, see Fig. 5). Participants were especially slower in the early phase of the trial, before rewards were displayed (β = 0.053 s, 95% CrI = 0.014 to 0.093 s, p(β = 0) = 0.24, BF_01_ = 0.32, p(β > 0) = 0.998, ER_+_  = 557.14), compared to the phase after the rewards were displayed (β = 0.011 s, 95% CrI = − 0.026 to 0.048 s, p(β = 0) = 0.97, BF_01_ = 28.12, p(β > 0) = 0.75, ER_+_  = 2.92). This slowing down in the early phase by ~ 50 ms was on average caused by a 25 ms longer reaction time but also a slower walking time by 25 ms for the first three steps (~ 2.3 m). To further analyze whether this slowdown was related to decision-making, we conducted an exploratory analysis between the difference in trial time with dual-tasking and differences in the reward effect and the swing leg effect with dual-tasking. There was no significant correlation between the slow down and the impact of multitasking on the reward effect (t(35) = 1.00, p = 0.49, 95% CI = − 0.16 to 0.47) or on the swing-leg effect (t(35) = − 0.71, p = 0.32, 95% CI = − 0.43 to 0.21). These findings suggest that participants’ changes in walking speed was not related to changes in decision-making.

## Discussion

In this paper, we set out to dissociate the processing of action-independent (i.e., reward) and action-dependent (i.e., motor cost) influences on value-based decision-making within a multitasking scenario. Prior research suggests that the cognitive weighing process of action-independent rewards is slow and limited by cognitive capacities^9^, whereas the weighing of action-dependent motor cost is faster and unconstrained by these limitations^19^. Hence, we hypothesized that the secondary task would predominantly impair reward processing, while it should not affect (or even increase) the impact of motor costs. Our results, however, seem to suggest something else. While we were able to replicate the influence of reward and motor cost on decision-making during walking^22,23^, our findings revealed that these decision-making processes were largely preserved under additional cognitive load. This persistence was, however, coupled with a slowing down in the early phase of a trial, culminating in an overall reduction of potential rewards due to exceeding the stipulated time limit.

There are a number of potential explanations for this result. One possible interpretation is that dual-tasking potentially impedes the selection of an optimal walking speed^39,40^. The additional cognitive load might have constrained the participants’ ability to initiate and control walking speed within the time constraint. This interpretation aligns with recent insights underscoring the reciprocity between cognitive tasks and motor actions, suggesting that motor processes might be more intertwined with cognitive processes than previously assumed^6,41^. These so called “embodied decision theories” posit a stronger interplay between decision-making and motor functions pointing to shared decision-action attributes like spatiality and temporality^42,43^.

In the context of decision-making, two primary implications arise. On the one hand, it is conceivable that the change in early walking behavior by a secondary task is unrelated to the value-based decision process. In this light the missing effect on decision-making would point to an automation of the weighing between reward and motor cost. Indeed, our current task was unambiguous, and often led to decisions with high success rates (participants consistently choosing the higher reward). This may imply that the task's reward system could have fostered a stimulus–response automation, where participants quickly identify and respond to the higher reward without extensive cognitive evaluation of value^44,45^. Participants had to choose between points, which, despite showing a gradual increase in influence on decision-making for higher rewards, might not fully capture the complexity of more nuanced value-based decisions. For instance, choosing between a banana and a peach, each with a personalized value, could engage a more demanding cognitive process. Therefore, while our results suggest an automated process in decision-making, they might be limited in their applicability to scenarios involving less salient or more abstract value-based decisions. Additionally, the modality compatibility between the tasks in our study deserves attention. The value-based decision task was visual-pedal, while the secondary sound task was auditory-verbal. Previous multitasking research indicates that dual-task interference is often minimized when tasks have different but compatible modality mappings^46^. This compatibility might partially account for the lack of a significant impact on decision-making observed in our study.

On the other hand, shared cognitive resources might impact both motor behavior and decision-making, indicating that individuals might modulate their walking speed to maintain decision accuracy or pitch identification accuracy. This introduces the notion of resource scheduling and puts emphasis on the speed-accuracy trade-off. That is, under multitasking demands, extending decision time may be a compensatory mechanism to maintain accuracy^47–49^. In other words, the delay observed in our participants' walking speeds might be a manifestation of participants’ strategy to judiciously allocate cognitive resources^50^, prioritizing accuracy over speed within a cost–benefit framework^51^. While we cannot rule out that the slowdown was strategically related to the decision-process, the results that participants slowed down before rewards were displayed combined with a lack of correlation between the slowing down and changes in decision-making challenge this view. Nonetheless, this situation complicates the interpretation of the observed absence of significant differences in decision-making and is further explored in a limitation section below.

Lastly, the temporal aspect of decision-making may also play a role^52^. The added cognitive task might have blurred participants' perception of time, leading to inadvertent exceeding of the allocated window, emphasizing the need for further exploration of time perception's interaction with decision-making^53^, especially when cognitive resources are partitioned between tasks. Similarly, increased focus on auditory cues might inadvertently synchronize step duration with the time intervals between sounds^54^. Given that these sound intervals lasted 625 ms—marginally longer than the average step duration of around 500 ms—this could contribute to a slower walking speed.

### Limitations

Even though we have reasoned against a potential speed-accuracy trade-off (see above), we acknowledge that we cannot completely rule out this alternative explanation. It is possible that participants preemptively slowed down immediately before the display of rewards in order to decrease their momentum during the decision-making process shortly after. Such a deliberate slowdown could afford them additional time or a more adaptable body state to make visual decisions based on rewards. In any case, this scenario would present a challenge to our original hypotheses as it is possible that dual-tasking affected decision-making if this slow-down would not be possible. This observation underscores the need for further empirical exploration, a topic explored in the subsequent section.

## Recommendations for future research and conclusions

Based on our findings, we provide a number of recommendations for future research to further elucidate the cost and reward considerations under dual-tasking.

To prevent the reward task from becoming automated through learning, thus bypassing additional valuation processes^21^, future studies could employ tasks necessitating more complex computations of reward values. Examples include decisions under risk^55–57^, decisions under uncertainty^58,59^, or less obvious choice options, like a preference for food items or faces^60^. One could also employ modality-compatible task mappings (e.g., a spatial secondary task) or limit the time rewards are explicitly displayed.

Additionally, distinguishing between influences on gait control and the potential for a speed-accuracy trade-off would be important. To achieve this, upcoming studies should consider controlling movement speed, for instance, by having participants walk to a set rhythm provided by auditory cues or by using a treadmill set to a constant speed. Additionally, these studies should evaluate baseline performance in the secondary task when performed in isolation^61^. If these temporal constraints have no effect on decision-making, but deteriorate performance in the secondary task, it would indicate that the interference primarily resides in walking speed regulation or the role of time in decision-making. Conversely, if decision patterns become more random concerning reward or motor cost, it would suggest a strategic allocation of resources to accommodate for the speed-accuracy trade-off, further elucidating how reward and motor cost are weighed under cognitive stress and the reliance on working memory.

In conclusion, our study suggests that dual-tasking does not directly modulate value-based choices but movement speed during walking, highlighting the complex interplay between cognitive and motor processes. These findings prompt further exploration and offer new avenues for research, emphasizing the need for more intricate tasks to unravel the nuanced relationship between decision-making and motor control in complex, multitasking environments.

### Supplementary Information

Supplementary Information.

## Data Availability

The datasets generated and analyzed during the current study are available in a OSF repository: https://osf.io/V85D7/ (10.17605/OSF.IO/V85D7).

## Code availability

Custom codes for the statistical analysis are available at: https://osf.io/V85D7/ (10.17605/OSF.IO/V85D7).
