# Supplementary material for: Dual-tasking modulates movement speed but not value-based choices during walking
Source: Sci Rep. 2024 Mar 15;14:6342. doi: 10.1038/s41598-024-56937-y (PMC10943095; doi:10.1038/s41598-024-56937-y)
Supplement: Supplementary file 1 — Supplementary Information. [file 41598_2024_56937_MOESM1_ESM.docx]

**Dual-tasking modulates movement speed but not value-based choices during walking**

Eric Grießbach^1,2*^, Philipp Raßbach^3^, Oliver Herbort^3^

& Rouwen Cañal-Bruland^2*^

^1^Department for Neurology, Johns Hopkins University Baltimore, MD, USA

^2^ Department for the Psychology of Human Movement and Sport,

Friedrich Schiller University Jena, Germany

^3^ Department of Psychology, Julius-Maximilians-Universität Würzburg, Germany

*Correspondence concerning this article should be addressed to Eric Grießbach ([griessbach.eric@gmail.com](mailto:griessbach.eric@gmail.com)) or Rouwen Cañal-Bruland ([rouwen.canal.bruland@uni-jena.de](mailto:rouwen.canal.bruland@uni-jena.de)).

Funding: This work was supported by the German Research Foundation (DFG) with two grants awarded to RCB (CA 635/4-1) and OH (HE 6710/4-1).

# Supplementary Information

## Methods

### Calibration and familiarization trials

To tailor task difficulty to each participant, we conducted calibration trials to individually set the starting position and time constraint. Participants were instructed to walk as quickly as possible between two points in the room for five trials. Based on prior studies^1,2^, the starting line was set at a distance 0.22 m shorter than the average walking distance of the first four steps. Also based on these prior studies, we added an additional 2.1 seconds, accounting for decision-making time, target orientation, and the 1.5 m distance to the target area. The onset of each calibration trial was defined as the moment one of the lateral malleoli markers surpassed a horizontal velocity of 0.1 m/s for a consecutive 0.125 seconds, determined by frame-to-frame positional derivatives.

### Determination of events during the experiment

The experiment employed real-time analyses to trigger the start of a trial, display rewards, and signal the trial's end. A trial commenced when four specific conditions were met continuously for 180 frames (1.5 seconds):

1. Six markers had to be visible within a designated area around the starting line. These markers were identified based on their expected positions (e.g., the left foot to the left of the right foot, toes positioned more forward than the lateral malleoli, and lateral malleoli more forward than the heels). 2. The foremost marker needed to be near the starting line, within horizontal and lateral tolerances of ± 0.05 m and ± 0.3 m, respectively. 3. The foot predetermined for the trial had to be positioned in front. 4. The marker on the malleolus had to remain stationary, indicated by a displacement below 0.004 m between consecutive frames.

The timing for displaying rewards was determined by kinematically estimating the moment of foot contact with the ground, known as "touch-down," during walking. Rewards were shown if a touch-down occurred behind a predefined reward trigger line, which was positioned to detect the step just before entering the central zone based on previous experiments. To identify each touch-down, we calculated the maximum horizontal distance between the heel marker on the swing leg and the lateral malleolus marker on the stance leg^3^. For the real-time analysis, this maximum distance was defined as the moment when the horizontal distance between two consecutive frames switched from increasing to decreasing. To prevent multiple detections for a single touch-down, the analysis was paused for 0.125 seconds after each touch-down. A trial was finished if more than four markers were identified in the target area, indicating that at least one foot had reached it. To verify if participants completed the task within the allotted time, the trial duration was measured using MATLAB's internal stopwatch functions ("tic," "toc") as the time between initiation of the trial and trial completion.

To ascertain which foot stepped into the central zone, we visually inspected the kinematic data after finishing the experiment. This was particularly important for trials where rewards were displayed late (the step was already in the central zone), the trial was incomplete, or kinematic data was missing. Such instances were rare and typically occurred due to marker loss or detection issues.

### Sensitivity and specifity

To evaluate participants' engagement with the secondary sound pitch classification task, we calculated both sensitivity and specificity. Sensitivity was assessed by measuring the true positive rate for correctly identifying high-pitched tones, while specificity was gauged through the true negative rate for accurately recognizing low-pitched tones. We assumed that tones not identified as high-pitched were considered low-pitched by the participants. To ascertain whether participants were actively attending to the task, we conducted binomial tests using a random sampler, set to the true proportions of high and low pitches (1/3 and 2/3, respectively).

### Statistical analysis

In our Bayesian model, we employed informative priors for the reward effect and weakly informative priors for the remaining parameters. The selection of these priors was guided by prior predictive checks, targeting a uniform distribution for equal rewards (50/50) and a skewed distribution favoring the side with higher rewards (60/40 and 40/60). Based on a similar study we conducted^4^, we used a normal distribution with a mean of 2 and a standard deviation of 1 for the reward effect. For the intercept, a normal distribution with a mean of 0 and a standard deviation of 1 was used. All other regression coefficients were modeled using a normal distribution with a mean of 0 and a standard deviation of 0.5. The standard deviation for all random effects followed an exponential distribution with lambda set at 2.

For model fitting, we utilized brms^5^, which operates on the Stan platform. Stan employs a Markov Chain Monte Carlo (MCMC) algorithm to sample the posterior distribution. We generated 12 independent Markov chains, each containing 6,000 samples. The initial 2,000 samples served as warm-up, and only the subsequent 4,000 samples were used for posterior approximation. Convergence was assessed through visual inspection of the chains and the Rhat statistic, which consistently yielded values below 1.01. The effective sample size across all models was above 10,000 for all relevant main effects (detailed model analysis are available in the OSF repository).

## Results

### Sensitivity and specifity

To test whether particpants adhered to perform the secondary task, we analyzed the sensitivity and specifity of identifying the sound pitch. Figure S1 illustrates that, apart from one participant, the achieved sensitivity and specificity values exceeded random sampling with accurate proportions. More importantly, the significant sensitivity and specificity values for all but one subject (p < 0.01) strongly suggest a performance surpassing random guesses. In contrast, the outlier displayed p-values of 0.28 for sensitivity and 0.003 for specificity, suggesting a lack of engagement in the secondary task. This participant was consequently omitted from further analysis. Additionally, there was a strong correlation between sensitivity and specificity (r(36) = 0.90, 95% CI = 0.82 to 0.95, p < 0.001), indicating that participants typically approached the task without distinct strategies, such as purposefully identifying more or fewer high-pitched tones to enhance sensitivity or specificity.

| 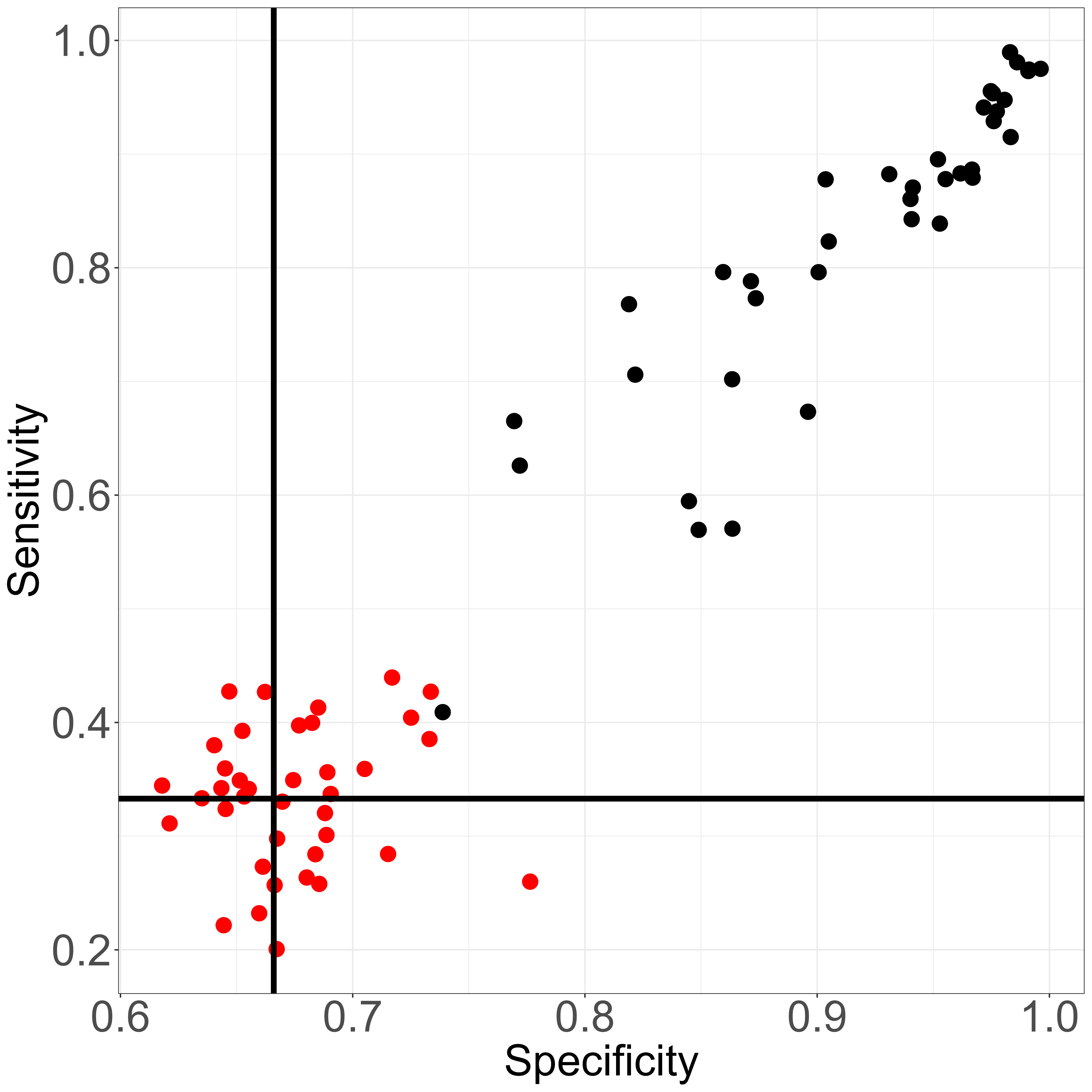  **Figure S1. Sensitivity and specificity of the pitch identification task.** Black dots represent individual participant values, and red dots signify random samples. Sensitivity measures the accurate identification of a high-pitched sound (true positive), and specificity gauges the correct rejection of a high-pitched sound when absent (true negative). Almost all participants, except one, demonstrated significant deviation from random samples, suggesting that they were attentive to the secondary task. |
| --- |

### Individual effect for decision-making

To bridge the gap between the model estimates presented in a log-odds scale in the main manuscript, we offer individual raw values and estimates converted to percentages on the response scale for contrasts relevant to our hypotheses (see figure S2).

| 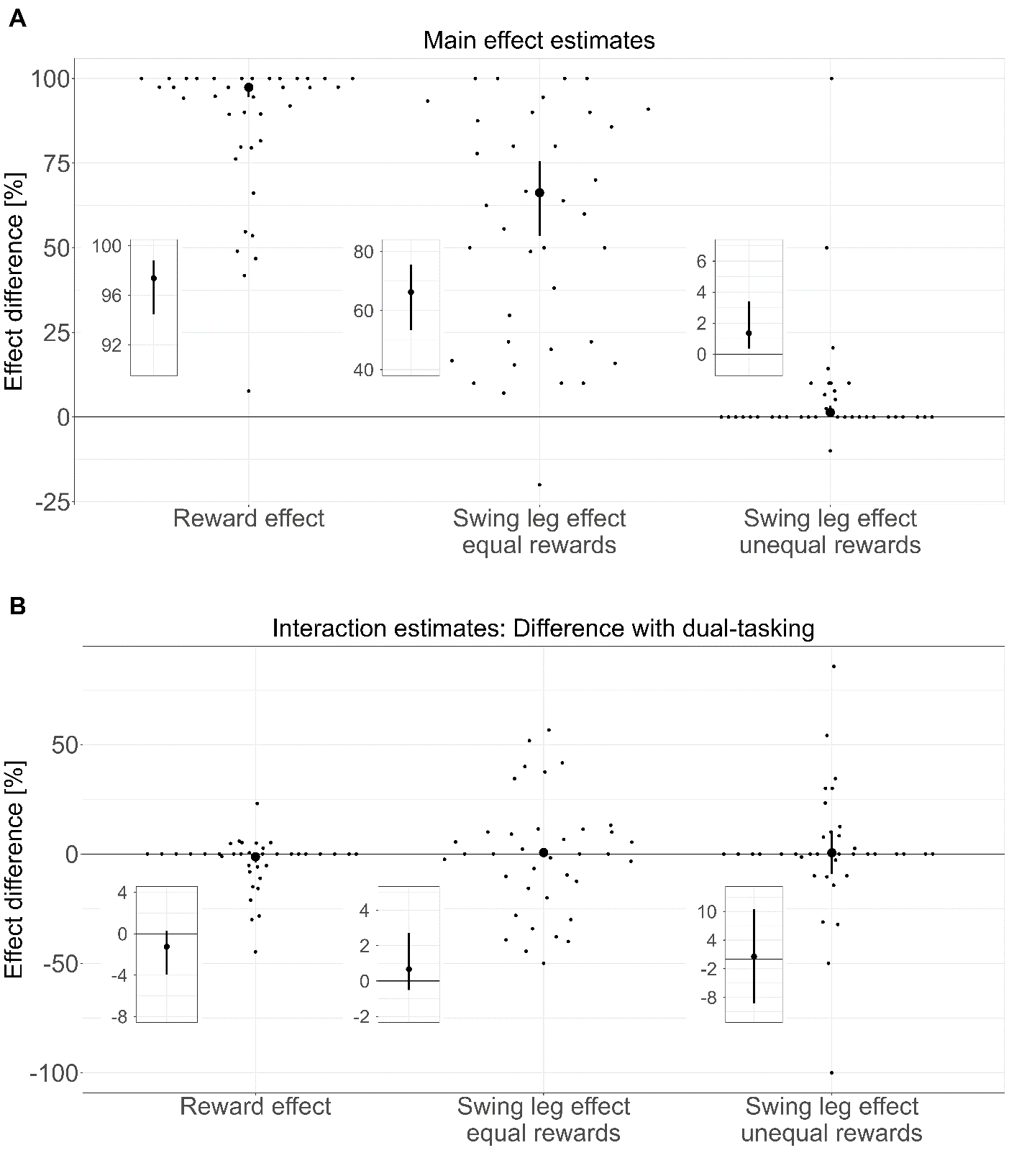  **Figure S2. Individual interaction effect and estimates for decision-making in response scale. A.** Main effect estimates on the probability scale for the RE and the SLE. The RE is the difference in the probability of going towards the right side given that higher rewards were on the right side vs. left side. Positive values for RE indicate a preference for the side with the higher reward. The SLE is the difference in the probability of going to the right side given a right vs. left swing leg in the central zone. Positive values for the SLE indicate a preference to choose the side congruent with side of the foot in the central zone, enabling a lateral step. Individual data points are raw percentage differences of each participant. For a more concise presentation, the figure displays a zoomed in plot of the mean estimates and 95% CrI. **B.** Interaction estimates (difference of difference) for dual-tasking vs single-tasking of the RE and the SLE. Negative values would indicate that the RE and the SLE decrease with dual-tasking. Individual data points are raw percentage differences of each participant. For a more concise presentation, the figure displays zoomed in plots of the mean estimates and 95 % CrI. DT = Dual-Tasking, RE = Reward Effect, SLE = Swing Leg Effect, ER = Equal rewards, UR = Unequal rewards. |
| --- |

# Literature

1 Grießbach, E., Raßbach, P., Herbort, O. & Canal-Bruland, R. Embodied decisions during walking. *J Neurophysiol* **128**, 1207-1223 (2022). <https://doi.org:10.1152/jn.00149.2022>

2 Grießbach, E., Raßbach, P., Herbort, O. & Cañal-Bruland, R. Embodied decision biases: individually stable across different tasks? *Experimental Brain Research* **241**, 1053-1064 (2023). <https://doi.org:10.1007/s00221-023-06591-z>

3 Banks, J. J., Chang, W. R., Xu, X. & Chang, C. C. Using horizontal heel displacement to identify heel strike instants in normal gait. *Gait Posture* **42**, 101-103 (2015). <https://doi.org:10.1016/j.gaitpost.2015.03.015>

4 Grießbach, E., Incagli, F., Herbort, O. & Cañal-Bruland, R. Body dynamics of gait affect value-based decisions. *Sci Rep* **11**, 11894 (2021). <https://doi.org:10.1038/s41598-021-91285-1>

5 Bürkner, P.-C. brms: An R Package for Bayesian Multilevel Models Using Stan. *J. Stat. Softw.* **80**, 1 - 28 (2017). <https://doi.org:10.18637/jss.v080.i01>
